# The Acquisition of Relative Clauses in Autism: The Role of Executive Functions and Language

**DOI:** 10.1007/s10803-023-06159-4

**Published:** 2023-10-28

**Authors:** Eleni Peristeri, Xanthi Kamona, Spyridoula Varlokosta

**Affiliations:** 1 School of English, Faculty of Philosophy, Aristotle University of Thessaloniki, Thessaloniki, 54124 Greece; 2 Department of Linguistics, Faculty of Philology, National and Kapodistrian University of Athens, Athens, Greece

**Keywords:** Autistic Disorder, Object and subject relative clauses, Relativised Minimality, Executive Function, Inhibition, Working Memory, Language

## Abstract

**Purpose:**

Relative clauses present a well-known processing asymmetry between object-extracted and subject-extracted dependencies across both typical and atypical populations. The present study aimed at exploring the comprehension of object and subject relative clauses as conceptualized by the Relativized Minimality framework in autistic children and in a group of age- and IQ-matched typically-developing children. The study also explored the way performance in relative clauses would be affected by the children’s language and executive function skills.

**Method:**

Relative clause comprehension was tested through a sentence-picture matching task and language was tested with a receptive vocabulary task. Executive functions were assessed through backward digit recall and a Flanker test.

**Results:**

Object relative clauses were harder to parse for both groups than subject relatives, while number mismatch between the moved object Noun Phrase and the intervening subject Noun Phrase in object relatives boosted both groups’ performances. Typically-developing children’s performance in object relatives was predicted by both language and executive functions, while autistic children failed to use language and did not systematically draw on their executive functions in object relative clause comprehension.

**Conclusion:**

The findings suggest that relative clause processing in autism follows a normal developmental trajectory, and that difficulty with parsing object relative clauses stems from reduced language and executive functions rather than deficits in the children’s morphosyntactic skills.

Autism is characterized by core difficulties in language and communication, even if fluent language has been achieved (Kjelgaard & Tager-Flusberg, 2001; Novogrodsky, 2013). These difficulties affect all linguistic levels, from syntax to discourse (Jarrold et al., 1997; Kjelgaard & Tager-Flusberg, 2001); however, there seems to be a high variability in language ability across autistic children, which is attributed to the high heterogeneity of autistic groups, as well as to the criteria for including children studies based on age, IQ, and the severity of autistic traits, among others. Previous research is mixed as to whether syntactic knowledge is deficient within autism more broadly or is only present when autism co-occurs with language impairment. Many studies have highlighted syntactic profiles in autistic children that are highly reminiscent of potentially undetected specific language impairment (SLI, currently known as Developmental Language Disorder) (Kjelgaard & Tager-Flusberg, 2001; Roberts et al., 2004; Tager-Flusberg, 2006; Tek et al., 2014; Wittke et al., 2017). Structures derived from syntactic movement, such as relative clauses, have been a testing ground for language impairment approaches to autism. Past cross-linguistic research has exhibited that autistic children fall behind their typically-developing (TD) peers in relative clause comprehension, and especially in object relative clauses (ORCs) (Durrleman et al., 2016, 2018) though other research (Schaeffer et al., 2018) has diverged showing no differences between TD and autistic children. In the current study, we aim at investigating the performance of autistic and age-matched TD children in subject and object relative clause comprehension, as well as the link(s) between the autistic children’s relative clause comprehension and cognitive skills, including inhibition and working memory. In what follows, we focus on the processing asymmetry that characterizes the comprehension of subject and object relative clauses in both autistic and TD children, as well as the factors (both linguistic and non-linguistic) that may contribute to this asymmetry. We also propose the study’s research hypotheses in light of studies relevant to each hypothesis.

Relative clauses present a well-known processing asymmetry between object-extracted and subject-extracted dependencies across both typical and autistic populations. Filler-gap dependencies have long been shown to be processed more easily in subject relative clauses (SRCs) [see (1)], where the gap is associated with the subject position of the relative clause than in ORCs [see (2)], where the gap is associated with the object position (Gordon et al., 2001; Holmes & O’Regan, 1981). This performance dissociation has been observed in TD (Corrêa, 1995; Kidd & Bavin, 2002) and autistic children (Durrleman et al., 2016), as well as in SLI (Friedmann & Novogrodsky, 2004; Martins et al., 2018), hearing impairment (Volpato & Adani, 2009), and adults with agrammatic aphasia (Grodzinsky, 1989). A factor relevant to this asymmetry, i.e. the advantage of SRCs over ORCs in comprehension, is the intervention effect created by the subject “the dancer” that disrupts the dependency between the moved object “the queen” and its gap after the verb “is holding” in (2). The intervention effect has been explained in terms of the Relativized Minimality (RM) framework (Friedmann et al., 2009; Rizzi, 2004), according to which, interference in long-distance dependencies is a function of the morphosyntactic feature overlap between the intervener and the extracted/moved element; as both the subject Noun Phrase (henceforth, NP) “the dancer” and the object NP “the queen” in (2) are lexically restricted, i.e. they share the [+ Nominal] syntactic feature, the subject NP functions as an intervener when the object NP moves to the left periphery, hindering the establishment of the syntactic dependency.

1. Show me the queen that ___ is holding the dancer. (no interference effect)
2. Show me the queen that the dancer is holding ___. (interference effect)

One core motivation for the processing difficulty characterizing ORCs is the similarity in the features of the moved element and the intervener. More specifically, studies that have confirmed the predictions of the RM account in children point out that the degraded acceptability of the dependencies involved in ORCs may vary as a function of the featural specification of the intervener and the extracted element. Specifically, ill-formedness is predicted to be stronger when the features of the intervener and the moved element match, than when features partially match (Friedmann et al., 2009) (also see Varlokosta et al. (2015) for evidence from Greek TD children). Importantly, according to RM, only morphosyntactic features triggering movement are relevant for the calculation of the featural overlap. In Adani et al.’s (2010) self-paced listening study with TD Italian-speaking children, center-embedded ORC comprehension was found to improve when the two NPs, i.e. the head of the RC “the waiter” and the subject of the RC “the boy” in (3), mismatched in number features, while there was no improvement when the two NPs mismatched in gender features, as in (4). In the examples (3) and (4) below, we provide morphological glosses, mainly limited to the features characteristic of the featural overlap between the two NPs, using the following abbreviations: NOM = nominative case, MASC = masculine, FEM = feminine, SG = singular, PL = plural.

(3) Il cameriere_NOM.MASC.SG_ che i ragazzi_NOM.MASC.PL_ salutano lavora qui.

“The waiter that the boys are greeting works here”.

(4) Il cameriere_NOM.MASC.SG_ che la ragazza_NOM.FEM.SG_ saluta lavora qui.

“The waiter that the girl is greeting works here”.

According to Adani et al. (2010), this asymmetry is attributed to the fact that number and gender behave differently because of their distinct morphosyntactic status in Italian: number plays an active role, i.e., it triggers movement, while gender does not (see also Bentea and Durrleman’s (2017) study for similar results in French). Belletti et al. (2012), on the other hand, have found that gender mismatch leads to higher ORC comprehension rates in Hebrew as compared to the number mismatch condition. The distinct performance patterns across Adani et al.’s (2010) and Belletti et al.’s (2012) studies have been captured by the differences in the syntactic status of the grammatical gender feature across the two languages; grammatical gender in Hebrew is morphologically expressed on the verb and attracts a NP to subject position, thus plays an active role in triggering movement, in contrast to Italian where grammatical gender is syntactically ‘inactive’. Durrleman et al.’s (2016) study with 4-, 6-, and 8-year-old French-speaking autistic and age-matched TD children showed that both groups were prone to feature similarity effects in ORC comprehension, since their comprehension performance in ORCs with number match between the moved element and the intervening subject significantly dropped as compared to the number mismatch condition. However, feature similarity in Durrleman and colleagues’ (2016) study seemed to play a role in the youngest, i.e. the 4 to 5 year-old age group only, further implying that parsing complexity effects originating from featural similarity in ORCs are overcome early in development in children with and without autism.

The overall results so far support the idea that distinctiveness amongst the lexical elements with respect to particular features can help lessen interference in retrieving the extracted element at its gap position in ORCs (Atkinson et al., 2016; Villata et al., 2016). This ameliorating effect critically relies on the syntactic status of the features, and more specifically, on their potential to incite syntactic movement. Also, the strength of RM effects depending on the nature of the morphosyntactic features of the NPs involved in the structures highlights the importance of studying the comprehension of ORCs in a wide array of languages with diverse grammatical properties, showcasing different morphosyntactic feature configurations. In this study, we aim to broaden our understanding of the effects of featural RM on the comprehension of relative clauses by investigating a much less studied language in the field of language processing in autism, i.e. Greek.

In recent implementations of the model (Corrêa et al., 2022; Guasti et al., 2018), the effects of RM on the comprehension of sentences involving filler-gap relations, such as ORCs, have been captured by other components of the parsing system, including interference and memory. Within the framework of cue-based retrieval parsing (Van Dyke, 2003), the integration of incoming words into sentence interpretation is limited by interference and memory constraints. With regards to interference, the more candidates match the moved NP, the greater the interference. In TD children (Adani et al., 2010; Belletti et al., 2012; Bentea & Durrleman, 2017) featural similarity between the intervener and the moved element leads to a degraded comprehension of ORCs, which is partially attributed to the fact that the moved NP is susceptible to interference from a NP that matches its features. As far as the memory factor is concerned, the displaced element “the queen” in (2) must be linked to its trace in post-verbal position, and until it is linked, it must be stored in working memory. These procedures incur processing costs in addition to establishing the dependency in the ORC. Inhibition of interference from subject NP-interveners along with working memory skills may be of key importance to the computation of syntactic dependencies in ORCs (Corrêa et al., 2022; Guasti et al., 2018).

Impairments in executive functions, including inhibition (Hill, 2004; Ozonoff & Strayer, 1997; Peristeri et al., 2021b; Robinson et al., 2009) and working memory (Kercood et al., 2014; Williams et al., 2005), have been extensively reported in school-aged autistic children. However, there are no studies examining RC comprehension and executive functioning in autistic children. The current study aims to fill this gap by investigating the performance of Greek-speaking autistic and age-matched TD children on RC comprehension within the context of the featural RM model, and also exploring possible relations between the children’s RC comprehension performance and their executive functions skills.

We ask the following research questions:

*Research question 1*. *Will autistic children exhibit a processing asymmetry between ORCs and SRCs, with the former structures being performed less accurately than the latter, and will autistic children’s comprehension performance be worse than their TD peers in ORCs and SRCs?*

Based on previous research (Durrleman et al., 2016) that has found significant complexity effects in relative clause comprehension in both young and older autistic and TD age groups, we expected that the autistic children in the current study would score lower in ORCs (vs. SRCs), however, we did not expect autistic children to score lower than their TD peers in ORCs.

*Research question 2. Will featural (mis)match in ORCs have an effect on autistic children’s comprehension performance?*

Based on previous research (Durrleman et al., 2016) showing that feature similarity effects in movement-derived structures manifest in young, 4 to 5 year-old autistic (and TD) children only, and since the current study included 7–11 year-old children, we did not expect feature similarity to play a significant role in either autistic or TD children’s comprehension of ORCs.

*Research question 3*. *Will autistic children’s inhibition and working memory skills be related to their comprehension of relative clauses?*

We hypothesized that the autistic children would show impairment in their inhibition and working memory skills as compared to their TD peers, and that their executive function deficits would affect their performance in RC comprehension. Specifically, we expected the executive function deficits to be mostly relevant to the autistic children’s comprehension of ORCs, where the establishment of long-distance dependencies between the moved NP and its trace strongly relies on the children’s inhibition and working memory skills.

## Methods

### Participants

The study included 42 children in total; 21 autistic children ranging in age from 7;6 to 11;1 years and 21 TD children ranging in age from 7;5 to 11;3 years. The autistic children were recruited from schools, and public and private diagnostic centers in Greece. They had received a diagnosis of autism from a licensed child psychiatrist according to the standard diagnostic criteria (American Psychiatric Association, 2000, 2013) and had full IQ scores (FIQ) at 80 or above, as measured through the standardized Greek version of the Wechsler Intelligence Scale for Children (WISC-III) (Wechsler, 1991; Georgas et al., 2003). TD children did not have a diagnosis of autism. They were recruited from public schools in Greece and no family history of learning disabilities or any history of language difficulties was reported for them by their parents. Written parental consent was provided for all participants. Details of the participants’ demographic characteristics are presented in Table 1. Descriptives were calculated using the statistical analysis software R (version 3.6.2; R Core Team, 2019). T-tests were performed to assess whether the two groups differed in age and IQ. The two groups did not differ significantly either in age, *t* = 1.777; *df* = 40; *p* = .083 or in their FIQ scores, *t* = 0.916; *df* = 40; *p* = .365.

**Table 1:** Experimental groups’ demographic information (age, IQ) presented as means (*SDs*).

	Group	
	Autistic	TD ^b^
N ͣ	21	21
gender (male)	16	14
age (in years)	9.6 (*1.2*)	10.2 (*0.9*)
age range	7;6–11;1	7;5–11,3
IQ	93.7 (*5.5*)	95.5 (*7.7*)
IQ range	88–108	83–110

ͣ = number

^b^ = typically-developing children

### Procedure

All children completed a receptive vocabulary test (the Peabody Picture Vocabulary Test (PPVT) (Dunn & Dunn, 1981; Simos et al., 2011), a backward digit recall test (Wechsler, 1991; Georgas et al., 2003), a flanker arrows task (Eriksen & Eriksen, 1974), and a relative clause (RC) sentence-picture matching task (adapted from Arfani et al., 2021).

The tests were administered in two sessions that took place on different days at the children’s home or school. Session 1 included the receptive vocabulary, the backward digit recall and the flanker arrows task, and session 2 included the RC sentence-picture matching task. To avoid stimulus order effects, the order of the two experimental sessions was counterbalanced, i.e. half of the children in each group (autistic, TD) received the tasks of session 1 first and the other half of the children received the tasks of session 2 first.

### Materials

#### Peabody Picture Vocabulary Test (PPVT)

The participants listened to a recorded word (verb, noun, adjective) and chose 1 out of the 4 images presented on a computer screen, the one that better matched the word they heard. The task consists of 173 words and ends automatically after the participant makes a specific number of errors. Regarding the tool’s psychometric properties (Simos et al., 2011), the PPVT has demonstrated very good-to-excellent test-retest reliability (*r* > .86 for total scores) and high convergent validity for similar constructs (WISC-III Vocabulary: *r* > .6) (Wechsler, 1991; Georgas et al., 2003).

#### Backward digit recall test

The maintenance and manipulation of information in WM was measured through the use of a backward digit recall task, which is part of the Greek version of the WISC-III (Wechsler, 1991; Georgas et al., 2003). The task consists of 8 conditions, starting from a 2-digit sequence and ending with a maximum of a 9-digit sequence. The length of each sequence of numbers increases by one digit when the participant repeats 4 consecutive sequences correctly in one condition. This task requires the participants to listen to a sequence of numbers spoken aloud by the experimenter and repeat them in reverse order. The task ends when the participants make 4 consecutive errors in one condition.

#### Flanker arrows task: Eriksen Flanker Task

In order to assess the children’s response inhibition through information processing and selective attention, the Eriksen Flanker Task was used. The task consists of 50 experimental trials in which the participants are presented with 5 arrows located in a box in a white background. Each trial consists of either congruent (→→→→→) or incongruent (→→←→→) arrow stimuli. The participants are instructed to focus on the direction of the middle arrow, while ignoring the rest of the arrows, by pushing as quickly as possible the “Z” button on the keyboard when the middle arrow points to the left and the “?” button when the middle arrow points to the right. Response accuracy and reaction times (RT) are automatically calculated. Dependent variables in the Flanker arrows task were accuracy and RTs in the congruent and incongruent conditions, as well as the conflict occurring at the visual interference level, which was calculated by subtracting RTs on congruent trials from RTs on incongruent trials (Christensen et al., 2018; Takezawa & Miyatani, 2005). Larger conflict values would indicate longer RTs for incongruent than congruent trials, further indicating inefficient inhibition of the interference inflicted by the surrounding arrows.

#### Sentence-picture matching task

**Fig. 1:**
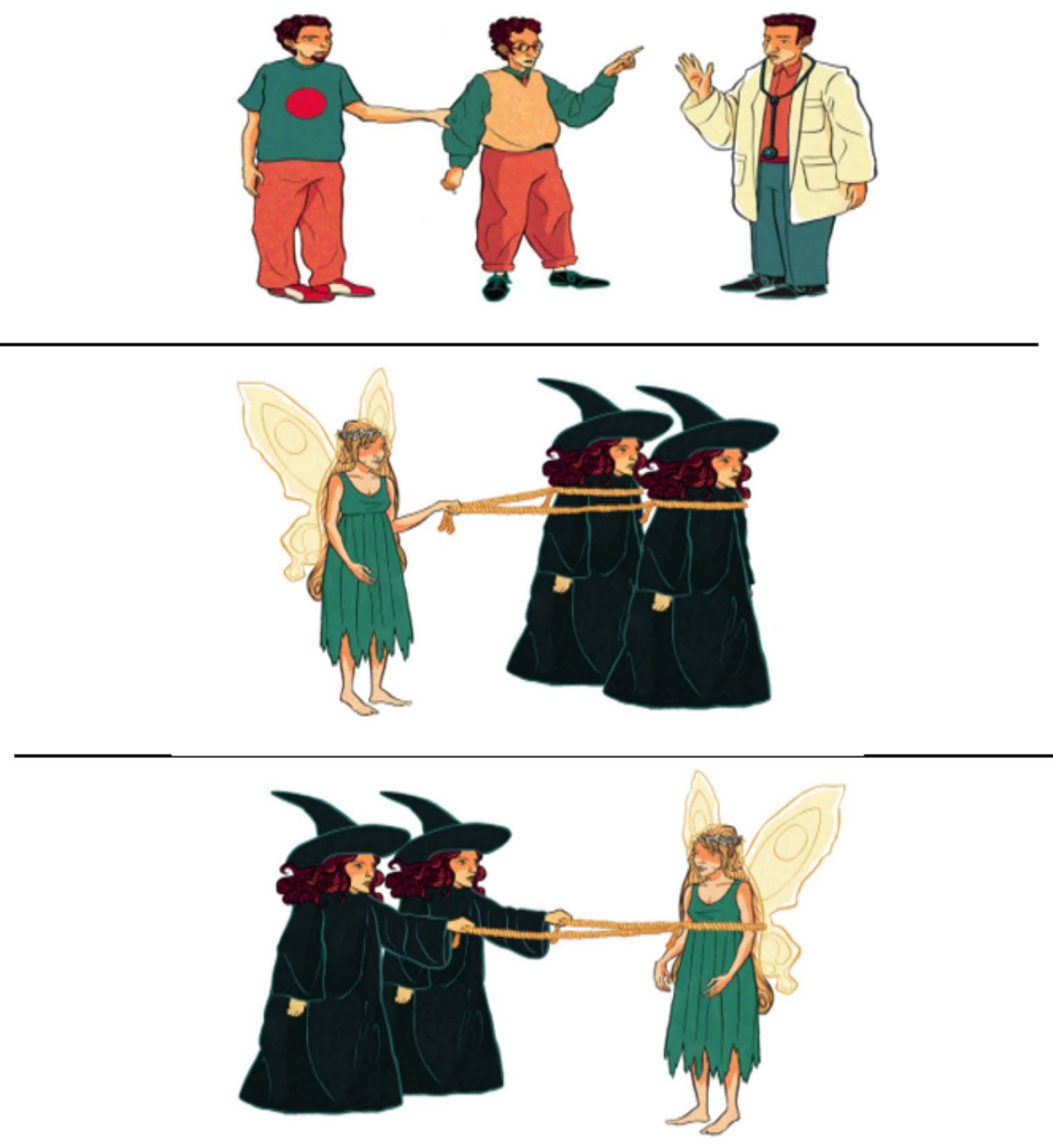
Example of a picture triplet trial in the relative clause sentence-picture matching task

To investigate the comprehension of subject and object right-branching RCs, a sentence-picture matching task was implemented adapted from Arfani et al. (2021). The participants were presented with three colored pictures on the screen of a computer and were asked to point to the picture that matched a clause they heard. In Fig. 1, the picture triplet assesses ORC comprehension in the gender match and number mismatch condition (Experimental sentence: “Show me the fairy that the witches are dragging”).

Crucially, besides the target picture, the second was a reversal foil portraying a reversal of thematic roles, while the third picture portrayed a different action and different characters from the ones encoded in the target sentence that the child listened to. All participants viewed the three pictures (1 target, 2 foils) in a different pseudorandomized order. The task consisted of seven conditions (see examples 5–11 below): (5) SRCs with gender and number match in the two NPs; (6) SRCs with gender match and number mismatch; (7) SRCs with gender mismatch and number match; (8) ORCs with gender and number match; (9) ORCs with gender match and number mismatch; (10) ORCs with gender mismatch and number match; and, finally, (11) active transitive sentences that served as fillers. There were 10 experimental sentences per condition, comprising a total of 70 experimental sentences.

(5) δikse mu ton vasilia pu akumpa ton anδra.

show me the _ACC.MASC.SG_ king_ACC.MASC.SG_. that is touching the _ACC.MASC.SG_ man_ACC.MASC.SG_.

“Show me the king that is touching the man”.

(6) δikse mu tin neraiδa pu trava tis maγises.

show me the _ACC.FEM.SG_ ferry_ACC.FEM.SG_. that is dragging the _ACC.FEM.PL_ witches_ACC.FEM.PL_.

“Show me the fairy that is dragging the witches”.

(7) δikse mu tin kopela pu hereta ton γiatro.

show me the _ACC.FEM.SG_ woman_ACC.FEM.SG_. that is waving the _ACC.MASC.SG_ doctor_ACC.MASC.SG_.

“Show me the girl that is waving at the doctor”.

(8) δikse mu ton mastora pu trava o papas.

show me the _ACC.MASC.SG_ craftsman_ACC.MASC.SG_. that is dragging the_NOM.MASC.SG_ priest_NOM.MASC.SG_.

“Show me the craftsman that the priest is dragging”.

(9) δikse mu ton δaskalo pu kitun i maθites.

show me the _ACC.MASC.SG_ teacher_ACC.MASC.SG_. that are looking the _NOM.MASC.PL_ students_NOM.MASC.PL_.

“Show me the teacher that the students are looking at”.

(10) δikse mu ti γiaγia pu akumba o δaskalos.

show me the _ACC.FEM.SG_ old lady_ACC.FEM.SG_. that is touching the _NOM.MASC.SG_ teacher_NOM.MASC.SG_.

“Show me the old lady that the teacher is touching”.

(11) o vasilias akumpa ton anδra.

the _ACC.MASC.SG_ king_NOM.MASC.SG_. is touching the_ACC.MASC.SG_ man_ACC.MASC.SG_.

“The king is touching the man”.

*Note*. MASC = masculine, FEM = feminine, NOM = nominative, ACC = accusative, SG = singular, PL = plural.

### Analysis Plan

All analyses were performed within the statistical analysis software R (version 3.6.2; R Core Team, 2019). For both the receptive vocabulary and the backward digit recall scores, we ran independent t-tests to compare the two groups. For the flanker task, mixed ANOVA analyses of Group (autistic, TD) by Congruency condition (congruent, incongruent) were conducted separately for accuracy rates and RT. Also, t-tests were run to compare the two groups on the flanker conflict cost, i.e. the RT difference between incongruent and congruent trials (Christensen et al., 2018; Takezawa & Miyatani, 2005). For the RC sentence-picture matching task, we computed accuracy rates for each of the seven conditions, i.e. active (ACT) transitives that served as the baseline; SRCs with gender and number match between the two NPs; SRCs with gender match and number mismatch; SRCs with gender mismatch and number match; ORCs with gender and number match; ORCs with gender match and number mismatch; and ORCs with gender mismatch and number match. The two groups were first compared on their accuracy rates in ACT clauses through t-test. We next ran repeated measures ANOVA analyses with Group as the between-subjects factor, and Clause type (SRCs, ORCs) and Match (feature match, feature mismatch on NPs) separately for gender and number as the within-subjects factors. Finally, linear mixed effects models were carried out to examine the influence of IQ, receptive vocabulary/PPVT scores, backward digit recall accuracy, and flanker metrics conflict cost as the independent variables on accuracy in each of the conditions of the RC sentence-picture matching task separately for each group (autistic, TD). Besides quantitative analyses, the children’s erroneous responses in the RC sentence-picture matching task were also recorded.

## Results

### PPVT, Backward Digit Recall and Flanker Test

Table 2 below displays the groups’ performance rates in the Greek standardized version of the PPVT, the backward digit recall and the flanker test. The t-test showed that the TD group had significantly higher receptive vocabulary scores than the autistic group, *t*(20) = 4.341, *p* < .001. In the backward digit recall test, the TD group had also significantly higher scores than the autistic group, *t*(20) = 3.266, *p* = .004. In the flanker test, the repeated measures analysis on accuracy revealed a significant Group effect, *F*(1, 40) = 5.591, *p* = .023, *η*^*2*^ = 0.11, which stemmed from the fact that the TD group was more accurate than the autistic group. There was also a significant Congruency effect, *F*(1, 40) = 22.296, *p* < .001, *η*^*2*^ = 0.14, which stemmed from the fact that accuracy in congruent trials was higher as compared to incongruent trials, and a significant two-way interaction between Group and Congruency, *F*(1, 40) = 17.736, *p* < .001, *η*^*2*^ = 0.03. To unpack the significant interaction we ran paired t-tests separately for each group. For the autistic children, congruent trials were more accurate than incongruent trials, *t* = 4.537; *df* = 20; *p* < .001. For the TD children, accuracy in congruent trials did not significantly differ from accuracy in the incongruent trials, *t* = 1.451; *df* = 20; *p* = .161. Regarding RT, there was a significant Group effect, *F*(1, 40) = 4.169, *p* = .048, *η*^*2*^ = 0.09, which stemmed from the fact that the TD group was faster than the autistic group. There was also a significant Congruency effect, *F*(1, 40) = 43.092, *p* < .001, *η*^*2*^ = 0.22, which stemmed from the fact that RT on congruent trials were faster than incongruent trials. The interaction between Group and Congruency in RT was not found to be significant, *F*(1, 40) = 0.036, *p* = .850, *η*^*2*^ = 0.01.

**Table 2:** Experimental groups’ mean performance rates (and *SD*s) in the PPVT-R, the backward digit recall and the flanker test

	Group	
	Autistic	TD^b^
N^a^	21	21
PPVT-R^c^	117.0 (*10.2*)	126.9 (*9.4*)
Backward digit recall	7.1 (*2.3*)	11.2 (*3.7*)
Flanker		
Accuracy in congruent trials	96.1 (3.7)	97.3 (2.3)
Accuracy in incongruent trials	92.7 (6.2)	97.1 (2.5)
RTs in congruent trials (in msecs)	992.2 (210.4)	872.0 (169.5)
RTs in incongruent trials (in msecs)	1011.3 (215.3)	890.1 (166.5)
Conflict cost (in msecs^d^)	19.1 (14.5)	18.1 (11.5)

^a^ = number

^b^ = typically-developing children

^c^ = Peabody Picture Vocabulary Test - Revised

^d^ = milliseconds

We further ran independent samples t-tests to investigate whether the autistic children would differ from TD children in each condition in either accuracy or RT. In accuracy, the two groups did not differ in congruent trials, *t* = 1.297; *df* = 40; *p* = .202, but TD children had significantly higher accuracy rates in the incongruent trials, *t* = 2.959; *df* = 40; *p* = .005. In RT, the autistic children were not significantly slower than their TD peers in either the congruent, *t* = 1.938; *df* = 40; *p* = .068, or incongruent condition, *t* = 1.941; *df* = 40; *p* = .068. Finally, the t-test showed that the autistic and TD group did not differ in the flanker conflict cost, *t*(20) = 0.166, *p* = .869.

### Sentence-Picture Matching Task

Table 3 displays the groups’ accuracy rates on the sentence-types of the RC sentence-picture matching task. We should note that besides 5 semantic errors produced by two out of the 21 autistic children, i.e. the two children picked the picture foil portraying different actions and characters, the rest of the erroneous responses in the autistic group consisted of thematic role reversal foils. Similarly, all errors in the TD group consisted of thematic role reversal foils.

**Table 3:** Experimental groups’ mean performance rates (%) (and *SD*s) in the relative clause sentence-picture matching task

	Group	
	autistic	TD^a^
N^b^	21	21
Active transitives	98.1 (*4.0*)	98.1 (*4.0*)
SRCs^c^_gender match_number match	97.1 (*4.6*)	100 (*0*)
SRCs_gender match_number mismatch	97.1 (*4.6*)	100 (*0*)
SRCs_gender mismatch_number match	99.5 (*2.2*)	98.1 (*4.0*)
ORCs^d^_gender match_number match	73.3 (19.3)	78.5 (17.4)
ORCs_gender match_number mismatch	85.2 (*10.8*)	92.4 (*7.7*)
ORCs_gender mismatch_number match	70.5 (*15.9*)	84.3 (*12.8*)

^a^ = typically-developing children

^b^ = Number

^c^ = subject relative clause

^d^ = object relative clause

The t-test showed that the two groups had the same accuracy scores in the ACT transitive condition, *t*(20) = 0.00, *p* = 1.00. The first repeated measures ANOVA analysis with Group as the between-subjects factor and Clause type (SRCs, ORCs) as the within-subjects factor for subject and object RCs with gender and number match features showed a significant Clause type effect, *F*(1, 40) = 61.604, *p* < .001, *η*^*2*^ = 0.43, which stemmed from the fact that accuracy in ORCs with gender and number match was considerably lower than their SRCs counterparts. There was no significant Group effect, *F*(1, 40) = 1.973, *p* = .168, *η*^*2*^ = 0.01, neither a significant two-way interaction between Group and Clause type, *F*(1, 40) = 0.171, *p* = .682, *η*^*2*^ = 0.01.

We next ran a repeated measures ANOVA analysis with Group as the between-subjects factor and Clause type (SRCs, ORCs) as the within-subjects factor for subject and object RCs with comparable feature (mis)match characteristics. With respect to SRCs and ORCs with gender match and number mismatch features, the analysis showed a significant Clause type effect, *F*(1, 40) = 34.026, *p* < .001, *η*^*2*^ = 0.31, which stemmed from the fact that accuracy in ORCs with gender match and number mismatch features was considerably lower than their SRC counterparts. The Group effect was significant, *F*(1, 40) = 13.283, *p* < .001, *η*^*2*^ = 0.08, since the autistic group scored lower than the TD group, yet, the two-way interaction between Group and Clause type was not significant, *F*(1, 40) = 1.640, *p* = .208, *η*^*2*^ = 0.02. The same analysis for subject and object RCs with gender mismatch and number match features showed a significant Clause type effect, *F*(1, 40) = 78.110, *p* < .001, *η*^*2*^ = 0.47, which stemmed from the fact that accuracy in object RCs with gender mismatch and number match features was considerably lower than their SRC counterparts. The Group effect was significant, *F*(1, 40) = 8.274, *p* = .006, *η*^*2*^ = 0.04, since the autistic group scored lower than the TD group, and the two-way interaction between Group and Clause type was also found to be significant, *F*(1, 40) = 9.875, *p* = .003, *η*^*2*^ = 0.06. To unpack the significant two-way interaction, we ran independent samples t-tests separately for subject and object RCs with gender mismatch and number match features. The two groups did not differ in SRCs, *t* = 1.430; *df* = 40; *p* = .160; however, the TD group scored significantly higher than their autistic peers in the ORCs with gender mismatch and number match features, *t* = 3.086; *df* = 40; *p* = .004.

We next ran paired t-tests between the feature (mis)match conditions within each Clause type (i.e. SRCs, ORCs) and within each group (autistic, TD). For the autistic group, accuracy in the SRCs with gender and number match features did not differ from either SRCs with gender match and number mismatch, *t* = 0; *df* = 20; *p* = 1.000, or SRCs with gender mismatch and number match features, *t* = 2.024; *df* = 20; *p* = .07. Similarly, SRCs with gender match and number mismatch did not differ significantly from SRCs with gender mismatch and number match features, *t* = 2.024; *df* = 20; *p* = .07. On the other hand, accuracy in the ORCs with gender and number match features was significantly lower than ORCs with gender match and number mismatch, *t* = 2.776; *df* = 20; *p* = .012, but similar to the accuracy in the ORCs with gender mismatch and number match features, *t* = 1.064; *df* = 20; *p* = .300. Finally, autistic children’s accuracy in ORCs with gender match and number mismatch features was significantly higher than ORCs with gender mismatch and number match features, *t* = 3.804; *df* = 20; *p* = .001.

For the TD children, accuracy rates in the SRCs across all feature (mis)match conditions, i.e. gender and number match, gender match and number mismatch, and gender mismatch and number match, were similar and near ceiling-level (range: 98.1-100%), so no statistical differences have emerged. Regarding ORCs, accuracy in ORCs with gender and number match features was significantly lower than ORCs with gender match and number mismatch, *t* = 4.221; *df* = 20; *p* < .001, but similar to accuracy in ORCs with gender mismatch and number match features, *t* = 1.826; *df* = 20; *p* = .083. Finally, TD children’s accuracy in ORCs with gender match and number mismatch features was significantly higher than ORCs with gender mismatch and number match features, *t* = 4.250; *df* = 20; *p* < .001.

The results of the linear mixed effects models for both groups are reported in Tables 4, 5 and 6 for the ACT transitives, SRCs and ORCs, respectively. The autistic children’s receptive vocabulary scores were significantly positively associated with their accuracy performance in ACT transitive clauses, SRCs with gender match and number mismatch features, and ORCs with gender mismatch and number match features. Flanker costs were significantly negatively related to comprehension accuracy in both ORCs with featural match in both gender and number, and in ORCs with gender match and number mismatch, while accuracy in ORCs with featural match in both gender and number was also found to be significantly predicted by the autistic children’s IQ scores. For the TD children, receptive vocabulary scores were significantly positively associated with their accuracy performance in ACT transitive clauses, ORCs with gender and number match, and ORCs with gender mismatch and number match features. The flanker conflict cost was (inversely) associated with both ORCs with gender and number match, and ORCs with gender match and number mismatch features. Finally, accuracy in backward digit recall was found to be significantly positively related to TD children’s comprehension accuracy scores in ORCs with gender match and number mismatch features.

**Table 4:** Potential predictors for autistic and typically-developing children’s accuracy in active transitive clauses

Predictor variables	Group									
	Autistic					TD^a^				
	Estimate	SE^b^	df^c^	t-Value	*p*	Estimate	SE	df	t-Value	*p*
IQ	7.245	1.850	1.553	3.916	0.088	1.316 × 10^− 4^	0.001	0.335	0.119	0.944
Peabody Picture Vocabulary Test	7.192	2.131	3.994	3.376	0.028*	0.020	0.008	15.670	2.367	0.031*
Backward digit recall	0.331	0.425	5.353	0.779	0.469	0.070	0.037	3.912	1.881	0.135
Flanker conflict cost	0.093	0.087	2.922	1.071	0.365	0.019	0.010	4.269	-1.865	0.131

^a^ = typically-developing children

^b^ = Standard Error

^c^ = difference

*p < .05

**Table 5:** Potential predictors for autistic and typically-developing children’s accuracy in subject relative clauses across the featural (mis)match conditions

Featural (mis)match conditions		Group									
	Predictor variables	Autistic					TD^a^				
		Estimate	SE^b^	df^c^	t-Value	*p*	Estimate	SE	df	t-Value	*p*
gender & number match	IQ	0.989	10.210	14.600	0.097	0.924	13.480	0.033	0.003	25.083	0.972
	Peabody Picture Vocabulary Test	1.607	5.910	13.807	0.272	0.790	0.289	0.012	3.386 × 10^− 4^	23.358	0.997
	Backward digit recall	0.003	2.512	1.572 × 10^− 4^	0.001	1.000	44.814	12.052	0.053	3.718	0.832
	Flanker conflict cost	-3.036	1.112	0.001	-2.730	0.993	− 0.064	0.024	0.002	-2.703	0.991
gender match & number mismatch	IQ	16.871	1.274	0.567	13.244	0.146	0.020	0.071	0.377	0.278	0.868
	Peabody Picture Vocabulary Test	1.395	0.742	10.462	1.879	0.088	0.037	0.084	15.125	0.447	0.661
	Backward digit recall	4.321	5.976	2.932	0.723	0.523	0.006	0.038	0.935	0.151	0.906
	Flanker conflict cost	-1.026	0.657	1.080	-1.563	0.349	-126.049	39.226	0.031	-3.213	0.895
gender mismatch & number match	IQ	10.490	4.608	0.691	2.276	0.343	0.051	0.028	13.280	1.829	0.090
	Peabody Picture Vocabulary Test	8.992	3.478	0.488	2.585	0.401	0.684	0.463	2.545 × 10 − 5	1.477	0.900
	Backward digit recall	4.967	1.977	0.211	2.512	0.612	0.007	0.009	10.623	0.836	0.422
	Flanker conflict cost	− 0.394	0.592	0.145	− 0.667	0.831	− 0.346	1.261	0.272	− 0.274	0.885

^a^ = typically-developing children

^b^ = Standard Error

^c^ = difference

*p < .05

**p < .01

***p < .001

**Table 6:** Potential predictors for autistic and typically-developing children’s accuracy in object relative clauses across the featural (mis)match conditions

Featural (mis)match conditions		Group									
	Predictor variables	Autistic					TD^a^				
		Estimate	SE^b^	df^c^	t-Value	*p*	Estimate	SE	df	t-Value	*p*
gender & number match	IQ	1.267	0.313	10.343	4.052	0.002**	6.602	3.297	10.851	2.002	0.071
	Peabody Picture Vocabulary Test	0.383	5.316	7.482	0.072	0.944	6.235	2.358	5.811	2.644	0.040*
	Backward digit recall	-1.533	1.347	10.634	-1.139	0.280	6.452	0.243	6.463 × 10^− 5^	26.545	0.999
	Flanker conflict cost	-15.850	5.659	3.979	-2.801	0.048*	-4.151	1.739	6.844	-2.387	0.049*
gender match & number mismatch	IQ	3.461	2.333	10.470	1.484	0.167	1.218	1.090	13.151	1.118	0.284
	PPVT	3.143	1.548	7.387	2.031	0.080	14.421	2.269	11.394	6.354	< 0.001***
	Backward digit recall	0.161	1.017	1.265 × 10^− 6^	0.158	1.000	3.205	0.079	16.946	40.440	< 0.001***
	Flanker conflict cost	-2.659	0.928	16.086	-2.866	0.01**	-0.585	0.151	16.986	-3.864	0.001**
gender mismatch & number match	IQ	-0.202	0.278	9.900 × 10^− 6^	-0.725	1.000	1.868	0.421	8.067 × 10^− 5^	4.440	0.999
	Peabody Picture Vocabulary Test	-4.933	2.090	0.203	-2.476	0.604	4.407	1.404	5.368	3.140	0.023*
	Backward digit recall	0.306	0.084	0.002	3.629	0.990	2.515	0.019	0.003	131.971	0.979
	Flanker conflict cost	− 0.072	0.207	5.858	− 0.348	0.740	-2.071	0.552	11.560	-3.752	0.003**

^a^ = typically-developing children

^b^ = Standard Error

^c^ = difference

*p < .05

**p < .01

***p < .001

## Discussion

In the present study, we sought to determine whether the autistic children would fall behind their TD peers in relative clause comprehension and whether they would exhibit a processing asymmetry between object-extracted and subject-extracted relative clauses. We also investigated whether the autistic children’s comprehension performance would be affected by the number or/and gender feature (dis)similarity between the moved element and the intervener in ORCs. Finally, we defined whether language, IQ and executive functions would be engaged by the autistic and the TD group during relative clause comprehension. We found that ORCs were harder to comprehend than SRCs for both experimental groups. Importantly, both autistic and TD children were affected by feature similarity interference, since ORCs with mismatch in their number features were performed better than ORCs with number feature identity. On the other hand, gender featural mismatch in ORCs had no facilitative effect for the comprehension performance of either group, which is consistent with the predictions of the featural Relativized Minimality framework (Friedmann et al., 2009; Rizzi, 2004; Villata et al., 2016) and its claim that interveners are less intrusive with theta role assignment when the moved element fails to agree in some feature with the intervener, provided this feature plays an active role in the movement-based derivation. Finally, we demonstrated that the TD group tended to systematically draw on language and executive function resources in ORC comprehension, while the autistic children failed to recruit language, and used executive functions selectively while performing in the ORCs of the sentence-picture matching task.

The first goal of the study was to determine whether the autistic and TD children would exhibit an asymmetry between SRCs and ORCs, and whether autistic children would score lower than their TD peers in any of the two clause types. The overall results of the sentence-picture matching task show that both experimental groups with and without autism exhibited an apparent asymmetry between SRCs and ORCs, since accuracy in the former clause type was significantly higher than accuracy in the latter type of clause. The specific results align with previous findings that due to their longer dependency and the subject intervening between the moved object and its gap, ORCs are more difficult to process than SRCs for both autistic (Durrleman et al., 2016) and TD children (Corrêa, 1995; Kidd & Bavin, 2002). Moreover, the autistic group’s comprehension performance did not significantly differ from the TD group in either clause type. Interestingly, a number of studies (e.g. Goodwin et al., 2015; Jyotishi et al., 2017; Su & Naigles, 2022) that have examined structures similar to relative clauses, such as Wh-questions, in English and Mandarin-speaking autistic children using intermodal preferential looking and play session paradigms have documented successful Wh-question comprehension and evidence of the object vs. subject asymmetry across the autistic groups, further implying that autistic children do not differ from their TD peers in grammatical knowledge. In the current study that has employed a static sentence-picture matching task, both autistic and TD experimental groups seemed to be performing above chance across all experimental conditions, which further implies that the autistic group had the core grammatical knowledge to interpret even the most difficult relative clauses in the task, namely, ORCs. Importantly, the errors committed by the autistic children almost exclusively consisted of thematic role reversal foils, in other words, the predominant deviant response that they adopted was to interpret an ORC as a SRC. This pattern of errors excludes lexical-semantic deficits or/and attentional biases stemming, for example, from autistic children’s propensity to focus on irrelevant details that would draw their attention away from the target picture (Baron-Cohen et al., 2009; Peristeri et al., 2020).

Another goal of the current study was to investigate possible feature similarity effects in the autistic children’s comprehension performance in ORCs. Crucially, number mismatch in ORCs yielded better performance than other types of ORCs for both the autistic and the TD group, which suggests that the autistic children were capable of exploiting morphosyntactic differences in terms of number information on the NPs, and that feature similarity affects ORC processing in a selective way depending on the nature of the morphosyntactic features involved in the derivation (Belletti et al., 2012; Friedmann et al., 2009); like in Italian and French, it is the number feature in Greek that triggers movement, thus, it has an active role in the establishment of syntactic dependencies, while gender does not (see also Alexandri et al., 2018; Arfani et al., 2021). Importantly, the gender mismatch condition in the current sentence-picture matching task did not significantly improve ORC comprehension in either of the two groups, suggesting that dissimilarity between the moved element and the intervener in terms of a syntactically inactive feature, such as gender, is insufficient to produce facilitation of theta role assignment (Angelopoulos et al., 2022). Previous research (Durrleman et al., 2016) evaluating feature similarity in ORCs in French has reported null effects for 6 and 8 year-old autistic children, who were age- and IQ-matched with TD children. The discrepancy between the results of the current and previous research (Durrleman et al., 2016) could reflect morphophonological differences between the two languages; number marking in Greek is morphologically manifested on NPs’ and verbs’ inflectional endings which are both audible to the listener, in contrast to French in which the morphological difference between singular and plural in the verbal inflection is always inaudible. It may be that the processing of features relevant to movement-based operations, such as the ones involved in ORCs, is facilitated by the audibility of these features, and that the feature similarity effect in the comprehension of ORCs is more pronounced when verbal inflectional endings are audible. However, further cross-linguistic research in this area is warranted to draw any definite conclusion.

The final goal of the study was to identify and compare the processing resources, including IQ, language and executive functions that the two experimental groups have drawn upon while performing in the sentence-picture matching task. Though TD children tended to use both language and executive functions, more specifically, inhibition, while performing in the ORCs across all the three feature (mis)match conditions of the task, the autistic children mainly used IQ and inhibition while parsing ORCs with feature identity, and inhibition while parsing ORCs with number feature mismatch. Furthermore, the autistic children failed to use any resources while processing ORCs with gender mismatch features.

It seems that each ORC condition loaded differently on the autistic children’s available resources depending on the featural make-up of the NPs in the ORCs. In the number mismatch condition at which the autistic children exhibited their highest accuracy level (85.2%), the interpretation of the clauses was not demanding probably due to the number mismatch facilitating effect, so only inhibitory skills were recruited. On the other hand, the performance of the autistic group has dropped by approximately 8% in the ORCs that involved gender and number similarity, which implies that the specific clauses represented a rather demanding condition because of the featural identity, thus, required more processing resources, namely, both IQ and inhibitory skills, relative to the number mismatch condition. The finding that the autistic children with higher IQ scores exhibited higher comprehension accuracy in ORCs with gender & number identity as compared to their peers with lower IQ scores seems to agree with the study by Durrleman and colleagues (2016), who also found that the autistic children’s ORC comprehension ability developed as a function of the children’s non-verbal reasoning skills. Intelligence appears to be an important factor regulating autistic children’s performance in language processing tasks, especially in syntax, and needs to be further explored to elucidate its function as compensating for language impairments in autism (Peristeri et al., 2021a, b, 2022). Finally, we obtained a null effect for the ORCs involving gender mismatch (and number feature match), which was the only condition at which the autistic group scored significantly lower than their TD peers. It seems that gender feature mismatch affected the recruitment of processing resources, possibly because of the autistic children’s reduced ability to comprehend the specific clauses.

Taken together, the results of the present study show that the autistic and TD children patterned alike in the processing of relative clauses, in that ORCs were more demanding than SRCs. Crucially, the experimental groups’ performance in ORCs with number feature mismatch between the extractee and the intervener was qualitatively the same, since both TD and autistic children experienced weaker interference from illicit antecedents as compared to the number feature matching conditions, which strengthens the predictions of the featural RM framework (Rizzi, 2004). Our study further showed that the autistic children’s failure to efficiently cope with featural overlap in ORCs may stem from reduced executive function, and especially, language resources, which were systematically recruited by their TD peers. The overall findings imply that the challenges posed by ORC processing in autism may not relate to their morphosyntactic knowledge per se, but to the children’s reduced ability to harness language resources and executive functions. These findings further suggest that teaching practices targeting language comprehension in autistic children may be ameliorated by placing emphasis on enhancing the students’ executive functions, which may in turn have a positive effect on their language comprehension skills.

Although the results of the current study provide evidence in favor of preserved grammatical skills in autistic children and their reliance on executive function skills to perform successfully in ORC comprehension, our findings should be replicated by follow-up studies given the following limitations. For one, the results need to be replicated with a larger sample of autistic children having a more narrow age range to control for possible heterogeneity owing to the children’s broad age range (7–11 years). Also, the sentence-picture matching task of the current study posed both language and joint attention demands to the children, which could be a concern for comparing TD and autistic groups given the known joint attention challenges in autism (Bruinsma et al., 2004). It is possible that the comprehension of ORCs might be modulated by other factors to those explored here, for example, reduced ability to coordinate with the examiner while performing the sentence-picture matching task. Thus, it might be of interest in future research to examine attentional skills alongside relative clause comprehension, so as to uncover other potential sources of relative clause performance in autistic individuals.

## Data Availability

Raw data were generated at the Psycholinguistics and Neurolinguistics Lab of the Faculty of Philology of the National and Kapodistrian University of Athens, Greece, and are at a drive repository (https://docs.google.com/spreadsheets/d/1ofSyJyTVHKC_NmWBb0aPU-RVvKNVfeOJ/edit#gid=1553539072). Derived data supporting the findings of this study are available from the third author [Spyridoula Varlokosta] on request.
